# A Rapid and Efficient Strategy for Quality Control of Clinopodii herba Encompassing Optimized Ultrasound-Assisted Extraction Coupled with Sensitive Variable Wavelength Detection

**DOI:** 10.3390/molecules27144418

**Published:** 2022-07-10

**Authors:** Yao Liu, Xiaojun Song, Xuebin Shen, Yuangen Xiong, Li Liu, Yuexi Yang, Sihui Nian, Limin Liu

**Affiliations:** 1 School of Pharmacy, Wannan Medical College, Wuhu 241002, China; liuyao@stu.wnmc.edu.cn (Y.L.); tsongxj@wnmc.edu.cn (X.S.); sxbchn@wnmc.edu.cn (X.S.); liulilili@stu.wnmc.edu.cn (L.L.); yx0603@stu.wnmc.edu.cn (Y.Y.); 2 Institute of Modern Chinese Medicine, Wannan Medical College, Wuhu 241002, China; 3 Anhui Pharmaceutical Co., Ltd., Huiyinbi Group Co., Ltd., Lu’an 237202, China; xyg19640806@163.com; 4 Anhui College of Traditional Chinese Medicine, Wuhu 241000, China

**Keywords:** Box-Behnken design, clinopodii herba, quality evaluation, ultrasound-assisted extraction, variable wavelength detection

## Abstract

Clinopodii herba is a folk herbal medicine for treatments of hemorrhagic disorders. However, there is not even a quantitative standard for clinopodii herba deposited in the Chinese Pharmacopoeia. The development of a strategy for rapid and efficient extraction and simultaneous detection of multiple components in clinopodii herba is therefore of great value for its quality evaluation. Here, a variable wavelength strategy was firstly applied to quantity multiple components by segmental monitoring by UHPLC with diode array detector following ultrasound-assisted extraction. The parameters of ultrasound-assisted extraction were optimized using single factor optimization experiments and response surface methodology by a Box–Behnken design combined with overall desirability. Subsequently, a rapid, efficient, and sensitive method was applied for simultaneous determination of eleven compounds, which represented the major and main types of components in clinopodii herba. Moreover, the performance of the validated method was successfully applied for the quality control of various batches of clinopodii herba and provided sufficient supporting data for the optimum harvest time. The Box-Behnken-optimized ultrasound-assisted extraction coupled with variable wavelength detection strategy established in this work not only improves the quality control of clinopodii herba, but also serves as a powerful approach that can be extended to quality evaluation of other traditional Chinese medicines.

## 1. Introduction

Traditional Chinese medicines (TCMs), an essential part of the healthcare system in many Asian countries, are becoming more and more popular around the world due to their reliable clinical effect in treating chronic and complex diseases [1,2,3]. The sophisticated chemical constituents are the bottleneck for its modernization. In most previous studies, only one or few marker constituents were considered for quality control, and the evaluation of overall efficacy and complex constituents of TCMs was far from enough [4,5,6]. Therefore, developing a rapid, efficient, and holistic quality control method of TCMs is urgent and valuable. Clinopodii herba (CH), which is attached to the *Lamiaceae Clinopodium* annual herbs of *Clinopodium polycephalum* (Vaniot) C.Y.Wu et Hsuan and *Clinopodium chinense* (Benth.) O. Kuntze, is a folk herbal medicine for various causes of hemorrhagic disorder, and the compounds of CH were classified into three primary categories: triterpenoid saponins, flavonoids, and phenylpropanoids [7,8,9,10,11]. Among these components, triterpenoid saponins showed hemostatic [12,13], cardioprotective [14], and anti-inflammatory activity [15]. Moreover, flavonoids exhibited antioxidant [16], immunoregulative [17], anti-inflammatory, and cardioprotective effects [18], while phenylpropanoids presented MMP-2 inhibitory activity and hypoglycemic effect [7]. Until now, there has been no efficient method of content determination applied for overall quality control of CH, only a thin layer chromatography identification test in the current Chinese Pharmacopoeia (2020 edition) [19]. Therefore, it was imminently necessary to develop a rapid and efficient quantitative method to determine as many bioactive compounds as possible to evaluate the holistic quality of CH.

Currently, one main method used for quality control of CH is quantitative determination of a single or a limited number of active components or marker compounds, such as triterpene saponins or flavonoids [20,21,22,23,24]. TCMs are considered to exert curative effects through the synergistic effect of multiple ingredients, multiple targets by multiple approaches [25]. Thus, a comprehensive strategy should consider not only the amount of active bioactive compounds, but also the structural types of the main ingredients for the holistic quality control of CH.

Moreover, extraction is very crucial for developing a rapid and efficient strategy. In this sense, ultrasound-assisted extraction (UAE) is considered as a promising approach to supersede the conventional methods because of the advantages of easy operation, high extraction efficiency, low solvent consumption, and short extraction times [26,27], which make it a quick and efficient approach for the pre-concentration of analytes from complex matrices. The efficiency of UAE is often influenced by the factors of extraction time, solvent concentration, solvent to solid ratio and ultrasonic power, so it further necessary to be optimized [28,29]. Response surface methodology (RSM) is a valid mathematical and statistical method to optimize the extraction process [30,31], which can investigate not only the single variables, but also the interaction between variables. Box–Behnken design (BBD), which is an effective tool of RSM, has been widely used for the optimization of experiments [32].

With the advantage of shorter analysis time and increased peak resolution, ultra-high performance liquid chromatography (UHPLC) was applied to determine multiple types of components in TCMs with diode array detector (DAD). Here, we established a strategy combining BBD-optimized UAE and UHPLC for rapid, efficient, and simultaneous determination of eleven bioactive compounds in CH, including three main structural types of chemical components (one triterpene saponins, one phenylpropanoids and nine flavonoids). The UAE parameters, which include proportion of aqueous methanol, liquid to solid ratio and extraction time were optimized with the single factor optimization experiments, and eleven bioactive compounds from CH were extracted by three variable and three level BBD-designed RSMs combined with an overall desirability (OD) value. Our study has demonstrated that the established strategy based on BBD-optimized UAE and variable wavelength detection using UHPLC–DAD provides a promising approach to assist the quality control of TCMs.

## 2. Results and Discussion

### 2.1. Selection of Markers of Quality Control

To evaluate the quality of CH holistically and objectively, it was necessary to control the main and major types of bioactive components. Here, we constructed the ultraviolet full wavelength of the chemical fingerprint of CH by UHPLC-DAD in order to provide an overview on the constituents presented in CH- and eleven main components were identified by comparing with reference substances (cynaroside (**1**), narirutin (**2**), apigenin-7-*O*-*β*-D-glucuronide (**3**), rosmarinic acid (**4**), buddleoside (**5**), luteolin (**6**), isosakuranetin-7-*O*-rutinoside (**7**), naringenin (**8**), apigenin (**9**), buddlejasaponin IVb (**10**), and isosakuranetin (**11**)) (Figure S1). Compounds **1–3**, **5–8**, and **11** are the main flavonoids, compound **4** is the main phenylpropanoid and buddlejasaponin IVb (**10**) is a quality control ingredient in the current Chinese Pharmacopoeia (2020 edition) [19]. Among them, for example, apigenin (**9**) displays anti-inflammatory, anti-microbial and anti-cancer properties [33]. Therefore, comprehensively considering the content of components, the structural type, the availability of reference standards and their bioactivity in CH, the above eleven substances were selected as the marker compounds for holistic quality control of CH.

### 2.2. Optimization of UHPLC–DAD Conditions

**Figure 1:**
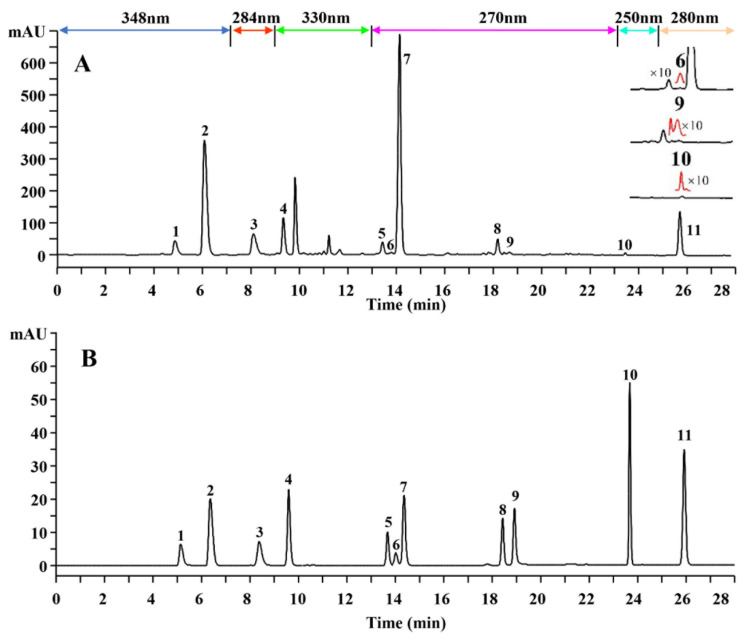
Chromatograms of (**A**) Clinopodium polycephalum sample and (**B**) mixed standard substances under segmental monitoring mode with HPLC-DAD. Standard substances representation: cynaroside (**1**), narirutin (**2**), apigenin-7-O-β-D-glucuronide (**3**), rosmarinic acid (**4**), buddleoside (**5**), luteolin (**6**), isosakuranetin-7-O-rutinoside (**7**), naringenin (**8**), apigenin (**9**), buddlejasaponin IVb (**10**) and isosakuranetin (**11**).

The chromatographic column, mobile phase, UHPLC parameters and detection wavelength were optimized to achieve a satisfactory separation and a good shape of the compound peaks. Among all the conditions, a suitable UV detection wavelength is a crucial factor for the analysis of TCMs with complex components. Universally, the single wavelength detection was adopted for quality control of TCMs, and the maximum UV absorption wavelength of target analyte was always chosen. However, this approach neglected weak signal peaks of some components with a weakly conjugated system and further affected the quality evaluation [34,35]. There are no available analytical methods for the detection of multiple types of constituents in CH simultaneously. To analyze them in one run, the feature wavelength was selected as detection wavelength for different structural types of components, and we divided the chemical fingerprinting into several segments according to the retention time of target compounds, and set specific UV absorption wavelength for each segment. The separation degree of each compound peak was the best on the Agilent ZORBAX Eclise Plus C18 column. The mobile phase was optimized, including organic phase and acidity, but only the acetonitrile–water containing 0.1% formic acid system was a relatively suitable resolution. Overall, the elution program was one of the most critical conditions that was optimized, which gave good separation of eleven standard substances. The representative UHPLC chromatograms of standard substances and the sample are shown in Figure 1.

Finally, according to their UV spectra (Table S1), for segmental monitoring based on variable wavelength detection, different detection wavelengths were performed for different periods of time: 348 nm for 0–7.3 min, 284 nm for 7.3–9.0 min, 330 nm for 9.0–13.0 min, 270 nm for 13.0–23.3 min, 250 nm for 23.3–24.7 min, and 280 nm for 24.7–28 min. For the different structural types, having different degrees of conjugation systems such that they are in the rising or descending part of the UV spectrum at a certain detection wavelength would affect the response of different components and further affect the accuracy of quantification [36]. Comparison of chromatogram B in Figure 1 and chromatogram d in Figure S1, the response of compound **11** is significantly enhanced in Figure 1B, which due to the maximum absorption of compound **11** at 280 nm and detection at 220 nm which is in the descending part of the UV spectrum (Table S1). Besides, although the other flavonoids and phenylpropanoids, both having strong conjugation systems so that prominent chromatographic peaks can be observed on the chromatogram (Figure 1B and Figure S1d), in order to improve the accuracy of quantification for markers and the corresponding absorption wavelengths are further re-selected for segmental detection. In this sense, triterpenoid, flavonoids and phenylpropanoids showed symmetrical peak shapes and relatively high intensity at the corresponding optimal detection wavelength. 

In terms of linearity, LOD, LOQ, stability, precision, repeatability and average recovery tests, the developed method was examined, and summarized results are presented in Table 1. All measurements followed the guidelines of Pharmacopoeia of the Peoples’ Republic of China, the first division of 2020 edition.

### 2.3. Method Validation

#### 2.3.1. Linearity, LOD and LOQ

The linearity of calibration curves for eleven compounds were established under six different concentrations using the peak area (*Y* axis) versus concentrations (*X* axis). As a result, good linearity correlation coefficients (*R*^2^) from 0.9995 to 0.9998 were obtained in the tested concentration ranges. Additionally, the sensitivity was evaluated by LODs and LOQs, and the results showed the LODs and LOQs of most compounds ranged from 4.6 ng/mL to 27.0 ng/mL and 9.1 ng/mL to 41.0 ng/mL, respectively, while for **6**, a higher LOD and LOQ at 140.0 ng/mL to 280.0 ng/mL were obtained, respectively, which may be due to the difference in chemical structure compared to other compounds.

#### 2.3.2. Precision, Repeatability and Stability

The precision method was applied to evaluate the repeatability of the method by six parallel repetitions of the same sample, and the RSD value was calculated for each compound with the range of 0.70–2.89%, which indicated this method was accurate. To verify repeatability, each standard solution was configured to six independent samples for parallel analysis, and variations were expressed by RSD. The stability of the standard solutions, which were stored at 4 °C, were detected during the analytical process within three consecutive days, and the RSD value of stability was lower than or equal to 2.48%, which showed that these samples have a good stability.

#### 2.3.3. Recovery

The recoveries were evaluated by the standards and samples mixed at 1.5:1, 1:1 and 0.5:1, respectively, and repeated three times (*n* = 9), the overall recoveries were 97.30–102.62% for eleven standards with RSDs ranging from 0.42% to 1.81%, which revealed the developed method was reliable.

### 2.4. Single Factor Experiment

#### 2.4.1. Optimization of the Proportion of Methanol–Water

The ratio of solvent was critical to obtain satisfactory efficiency for CH using UAE. Extraction by methanol–water was more efficient than methanol individually [37]. Extraction efficiency of methanol–water (40, 50, 60, 70, 80 and 90%, *v*/*v*) was investigated and the other parameters, including extraction time (30 min), liquid to solid ratio (50:1 mL/g, *v*/*w*) and ultrasonic power density at 2000 W/g were constant. Increasing the proportion of methanol–water ranging from 40% to 70% led to the increase in extract efficiency of total components (Table S2). A further increase in proportion beyond 70% caused decrease in response. Since various proportions showed differences in polarity, one solvent would be insufficient to extract the ingredients, hence, increasing the amount of methanol would break the cell membrane and promote the release of ingredients [38]. Therefore, the best extraction efficiency was obtained with the 70% methanol–water solution.

#### 2.4.2. Optimization of Liquid to Solid Ratio

The liquid to solid ratio also influenced the rate of all components from medicinal powder. As shown in Table S2, the OD value increased continuously as the liquid to solid ratio raised from 30:1 to 50:1, yet gradually decreased as the liquid to solid ratio was further increased, and the other parameters, including methanol–water solution (*v*/*v*, 70%), extraction time (30 min) and ultrasonic power density at 2000 W/g, were constant in this process. Therefore, 50:1 mL/g was selected as the optimal liquid to solid ratio.

#### 2.4.3. Optimization of Extraction Time

As shown in Table S2, when the extraction time was set within 35 min, the total extraction efficiency of 11 compounds was positively affected, and the maximum OD value was 0.873 ± 0.093, and the other parameters were kept constant as follows: 70% methanol–water solution (*v*/*v*), liquid to solid ratio (50:1 mL/g) and ultrasonic power density at 2000 W/g. As a result of increased time over 35 min, the contact surface between the solvent and the solid material would expand, further destroying cell walls and thus allowing the higher mass transfer. As observed, a further increase in extraction time resulted in decreased total extraction efficiency and inferred the possible reason is that the longer extraction time would cause the acoustic cavitation to completely destroy all plant cells, thereby promoting the release of insoluble substances and cytoplasm from the cells. Redissolving into the extraction solvent, thereby limiting the solubility and permeability of the solvent, and the potential for reabsorption of target components into broken plant particles, may also affect the yield of recovered compounds [39]. Finally, 35 min was selected as the optimal extraction time for the following study.

### 2.5. Optimization of UAE by RSM

#### 2.5.1. Model Fitting and Statistical Analysis

RSM is an effective mathematical model to optimize extraction processes through calculating the effect of each factor and their interactions using a Design Expert 12.0.3.0. As one of the tools of RSM, BBD was used for predicting the optimal experiment conditions based on the results of single factor experiments, a 17-run BBD was used to optimize the three variables, including the proportion of methanol–water (*X*_1_), liquid to solid ratio (*X*_2_) and extraction time (*X*_3_). Comparing with index summation, the OD method considers the comprehensive effect of each index and is more suitable for a multi-response system. Therefore, the OD values of eleven compounds were applied as an evaluation index. The experimental conditions and the response variable (OD value) are shown in Table 2. The associations were developed between response variable (*Y*, OD value) and variables by using multiple regression analysis according to the experimental data:

(1) $$Y=0.81-0.22X_{1}+0.2X2_{\;}+0.084X_{3}+0.012X_{1}X_{2}+0.076X_{1}X3_{\;}-0.014X_{2}X3_{\;}-0.33X_{1}^{2}\;-0.031X_{2}^{2}\;-0.069X_{3}^{2}$$

Variance analysis results were used to evaluate the effectiveness and adequacy of the fitting modes. A greater *F*-value and a smaller *p*-value imply more significant corresponding variables, and the model can be considered as significant when the *p*-value is less than 0.05. In this work, the *F*-value (*F* = 60.84) and *p*-value (*p* < 0.0001) revealed the model was appropriate to fit the experimental data (Table 3). Moreover, the lack of fit test can also be used to determine the significance of the model. The *p*-value of the lack of fit test (*p* = 0.0760) was higher than 0.05, indicating the model was significant. Meanwhile, the value of the determination coefficient (*R*^2^ = 0.9874) was close to the adjusted determination coefficient (*R*^2^_adj_ = 0.9711), which also confirmed the model was appropriate. In conclusion, the model was enough to navigate the actual relationship between the response and variables within the range of the experimental variables. The significance of each coefficient was checked by *p*-value, which explained the interaction between the variables. It can be seen that the coefficients of *X*_1_, *X*_2_ and $X_{1}^{2}$ were more significant (*p* < 0.0001). Similarly, the coefficients of *X*_3,_ *X*_1_*X*_3_ and $X_{3}^{2}$ were regarded as significant (*p* < 0.05), whereas the coefficients of *X*_1_*X*_2_, *X*_2_*X*_3_ and $X_{2}^{2}$ (*p* > 0.05) had no significant effect (Table 3).

#### 2.5.2. Analysis of the Response Surface

**Figure 2:**
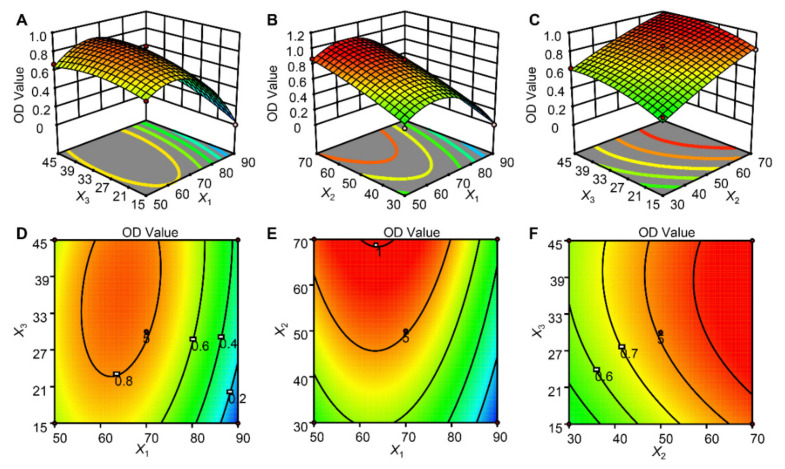
3D-response surface plots and 2D-contour plots showing the effect of different extraction parameters (*X*_1_: proportion of methanol–water , %; *X*_2_: liquid to solid ratio, *v*/*w*; *X*_3_: extraction time, min) on the response yield. Keeping *X*_2_ constant at 50 mL/g, *X*_3_ constant at 35 min, and *X*_1_ constant at 70% (*v*/*v*) for (**A**,**D**), (**B**,**E**) and (**C**,**F**), respectively.

A 3D response surface and contour plots were obtained by the Design-Expert, which illustrated the graphical relationship between response variable and the independent parameters (Figure 2). The shapes of the contour plots indicated whether the reciprocal interactions between the variables were significant. The interaction between the variables could be ignored in circular contour plots, however, the interaction between the variables could not be ignored in elliptical contour plots. In these three variables (proportion of methanol–water , liquid to solid ratio and extraction time), the effect of the relationship between two variables on the extraction efficiency was observed by keeping another variable constant at 0 level. 

#### 2.5.3. Analysis of the Response Surface

The interaction effect of the proportion of methanol–water (*X*_1_) and extraction time (*X*_3_) on the response value at a constant liquid to solid ratio is shown in Figure 2A,D. Experimental observations indicated that increasing the methanol–water concentration from 40% to 70% and extraction time from 5 min to 35 min indicated enhanced yields, yet further increasing the methanol–water concentration and time beyond these values indicated reduced yields. Their interaction was significant to affect extraction efficiency by UAE, as represented by the corresponding *p*-value and *F*-value of 0.0165 and 9.84, respectively. This was because ultrasound deduces acoustic cavitation and the fracture of plant cells which accelerated the penetration of the solvent into plant cells and dissolved the target constituents [39]. Longer extraction time would completely break all the plant cells by acoustic cavitation, therefore, the extraction yield would increase in a certain time [40]. However, completely fragmentized plant cells would also release various compounds such as insoluble and cytosolic substances to be redissolved into the extraction solvent, thus limiting the solubility and permeability of the solvent. Furthermore, the target components might be reabsorbed into the broken plant particles, which could also affect the yield of recovered compounds. In this sense, it would be expected that increased solvent concentration might increase the extraction yield. From Figure 2A, increased OD values were observed with an increased proportion of methanol–water from 40 to 70%. 

Adjusting the liquid to solid ratio can help change the solubility and equilibrium constant in the UAE system [41], therefore, the interactive relationship between the proportion of methanol–water and liquid to solid ratio was necessary to be evaluated. As can be seen from Table 3, the effect of the proportion of methanol–water (*F*-value = 160.00) was larger than the liquid to solid ratio (*F*-value = 138.76), and they had a significant effect on extraction efficiency. The OD value of the response variable was increased with the ascending liquid to solid ratio range from 30:1 to 50:1 mL/g, yet the value decreased as the liquid to solid ratio ascended afterwards. The OD value ascended firstly and then descended with ascending liquid to solid ratio by analyzing the 3D plot and 2D contour plot (Figure 2B,E). However, their interactive relationship was not significant, as shown by the corresponding *F*-value and *p*-value of 0.26 and 0.6426, respectively. Moreover, Figure 2C,F indicated a higher level of OD value to be obtained for the liquid to solid ratio ranging between 45 and 55%, regardless of the level of extraction time in UAE, illustrating the interactive relationship between extraction time and liquid to solid ratio was not significant. Although, the independent variables could significantly affect the extraction efficiency, however, their interaction was not significant, as shown by a high *p*-value (0.5802) and a small *F*-value (0.34), respectively.

The 3D analysis of RSM was performed based on the selected optimal conditions by single factor experiments. The results showed that the optimal parameters for UAE of CH were a methanol–water proportion of 64.63%, liquid to solid ratio of 70:1 mL/g and extraction time of 40.40 min. Considering the operability in actual situations, the optimal parameters were modified as follows: methanol–water proportion of 65.00%, liquid to solid ratio of 70:1 mL/g and extraction time of 40.00 min. Under this condition, the response variable (OD value) was 0.99 (Table S3), which was not significantly different from the predicted value (1.02) by the model. The results of the analysis confirmed that the BBD model was adequate and reliable for predicting the expected conditions. Consequently, these conditions were performed to determine the content of eleven compounds in samples.

### 2.6. Quantitative Analysis of CH

The developed and validated UHPLC-DAD method in this study was applied to quantitative analysis of major and main structural types of chemical components of CH in multiple batches of CH samples of different harvest periods. Considering the presence of these components in both *Clinopodium polycephalum* and *Clinopodium chinense* and their representativeness and accessibility of chemical components, finally, eleven compounds were selected as the markers including nine flavonoids (cynaroside, narirutin, apigenin-7-*O*-*β*-D-glucuronide, buddleoside, luteolin, isosakuranetin-7-*O*-rutinoside, naringenin, apigenin and isosakuranetin), one phenylpropanoid (rosmarinic acid) and one triterpenoid saponins (buddlejasaponin IVb), as shown in Table 4 and Figure S2. The samples analyzed show a remarkable difference in the total content of the eleven compounds that were noted in different growth periods of CH. As shown in Figure S3, their total content crest stage is June–July, which may be explained by the fact that the plants gradually mature and bear seeds, and the proportion of leaves gradually decreases from July to August (Figure S4), indicating the most appropriate harvest time is from June to July. Furthermore, there were differences not only in total content at different growth periods but also in the content of single components. The main chemical components are triterpenoid saponins and flavonoids in CH, among them, the content of flavonoids accounted for a large proportion of the total content. Figures S5 and S6 shown that the content of isosakuranetin-7-*O*-rutinoside in June and July was higher than that in August, and the content of isosakuranetin-7-*O*-rutinoside was obviously higher than that of rosmarinic acid.

In traditional cultivation, when CH flowers are gradually formed, the flowers and some leaves will gradually drop off and gradually form seeds, thus increasing nutrient consumption and reducing the chemical composition content of the whole plant. On this basis, we recommend an early (around July) best time to harvest the aboveground portion of corn.

## 3. Materials and Methods

### 3.1. Reagents and Materials

A total of 24 batches of samples (S1/June–S8/August) were collected from Lu’an city, Anhui Province in August 2021 and the sources are listed detailly in Figure S4 and Table S4. These samples were identified as *Clinopodium polycephalum* (Vaniot) C.Y.Wu et Hsuan by Prof. Sihui Nian, and the voucher specimens were deposited in School of Pharmacy, Wannan Medical College, Wuhu, Anhui Province. The plant samples were air-dried in shade and were then pulverized into a fine powder (50 mesh). The standard substances of cynaroside (**1**), narirutin (**2**), rosmarinic acid (**4**), buddleoside (**5**), luteolin (**6**), isosakuranetin-7-*O*-rutinoside (**7**), naringenin (**8**) and isosakuranetin (**11**) were purchased from Chengdu Desite Biotechnology Co., Ltd. (Chengdu, China), and standard substances of apigenin-7-*O*-*β*-D-glucuronide (**3**), apigenin (**9**) and buddlejasaponin IVb (**10**) were purchased from Pusi Biotechnology Co., Ltd. (Chengdu, China). The purity of standard substances was more than 99% determined by HPLC-DAD analysis (Figure S7 and Table S5). Acetonitrile and Methanol (HPLC grade) were purchased from Merck (Darmstadt, Germany). Formic acid (HPLC grade) was purchased from Roe Scientific Inc. (Newark, NJ, USA). Ultrapure water was acquired from a Milli-Q system (Millipore, Bedford, MA, USA).

### 3.2. Instrumentation and Chromatographic Conditions

UAE was performed with a KQ5200E ultrasonic cleaner (Kunshan Ultrasound Instrument Co., Ltd., Kunshan, China). UHPLC-DAD analysis of medicinal materials and preparations was performed on an Agilent 1290 infinity II UHPLC system (Agilent Technologies, Inc., Santa Clara, CA, USA) equipped with a 1290 DAD detector and a 1290 vial sampler using Agilent ZORBAX Eclise Plus C18 column (2.1 mm × 100 mm, 1.8 μm) coupled with an Agilent UPLC guard 3PK (2.1 × 5 mm, 1.8 μm). The mobile phases for UHPLC-DAD analysis were water containing 0.1% formic acid (A) and acetonitrile (B), and the gradient elution was 15% B (0–3 min), 15–18.5% B (3–4 min), 18.5% B (4–8 min), 18.5–24% B (8–10 min), 24% B (10–16 min), 24–30% B (16–17 min), 30% B (17–19 min), 30–38% B (19–20 min), 38–40% B (20–23 min), 40% B (23–25 min), 40–44% B (25–26 min) and 44% B (26–28 min) with a flow rate of 0.3 mL/min, and the column temperature was controlled at 30 °C. Meanwhile, the online monitoring wavelengths were 0–7.3 min (348 nm), 7.3–9.0 min (284 nm), 9.0–13.0 min (330 nm), 13.0–23.3 min (270 nm), 23.3–24.7 min (250 nm) and 24.7–28 min (280 nm). The injection volume was 3 μL.

### 3.3. Preparation of Solutions

#### 3.3.1. Preparation of Standard Solutions

The chemical standard solutions were prepared by dissolving eleven standard compounds (**1**, 1.43 mg; **2**, 10.88 mg; **3**, 1.99 mg; **4**, 1.95 mg; **5**,1.60 mg; **6**, 0.57 mg; **7**, 10.76 mg; **8**, 0.70 mg; **9**, 0.63 mg; **10**, 1.01 mg; **11**, 1.00 mg) in 10 mL 80% methanol–water solution and then diluted to appropriate concentrations for calibration curves. The standard solution was mixed and stored at 4°C. The calibration curves were prepared by diluting the mixed standard stock solution with 80% methanol–water solution at different concentrations.

#### 3.3.2. Preparation of Sample Solutions

An aliquot of 0.1 g powder was accurately weighed and suspended into 7 mL of 65% methanol–water solution in 25 mL stoppered conical flasks, then the mixture was subjected to ultrasonic extraction with a stationary ultrasonication power density (2000 W/g) at 30 °C for 35 min. After being cooled to room temperature, the crude extract was weighed again. The lost weight was supplemented with the same solvent (65% methanol), and then the solution was centrifuged at 4000 rpm for 8 min and the supernatant was filtered through a 0.22 μm membrane filter. All sample solutions were stored at 4 °C before analysis. Then, 3 μL of the solution was injected into the UHPLC–DAD system.

### 3.4. Ultrasound-Assisted Extraction of CH and Preparations

UAE was carried out in an ultrasonic cleaner at 2000 W/g ultrasonication power density. The CH powder (0.1 g) was put into a 25 mL stoppered conical flask and was extracted with methanol–water proportion (70%), liquid to solid ratio (50:1, *v*/*w*) and extraction time (35 min) at 30 °C for single factor experiments.

### 3.5. Experimental Design

#### 3.5.1. Single Factor Experimental Design

The effects of variables including the ratio of methanol–water solution (40–90%, *v*/*v*), liquid to solid ratio (30:1–70:1 mL/g) and extraction time (5–45 min) were selected, then the single factor experimental design was done by varying one factor at a time while keeping the others constant (Table S2).

#### 3.5.2. Box-Behnken Design

RSM was selected to optimize the conditions of processing technology. According to the results of single factor optimization, three variables including methanol–water concentration (*X*_1_), liquid to solid ratio (*X*_2_) and extraction time (*X*_3_) were selected and examined in three levels, then the BBD including 17 experiment runs was performed at random so as to evaluate the main effects of the factors for the optimization of the UAE parameters, as shown in Table 2 and Table 5. Based on the single factor experiments, the range of three independent variables were selected. The OD value of the content of buddlejasaponin IVb, isosakuranetin-7-*O*-rutinoside and the other compounds was taken as the evaluation index of the design experiments. A second-degree polynomial response surface model was used to evaluate the extraction efficiency, and the following formula was used for the calculation:

(2) $$Y=\beta_{0\;}+\overset{3}{\underset{i=1}{\sum }}\beta_{i}x_{i}+\overset{3}{\underset{i=1}{\sum }}\beta_{ii}x_{i}^{2}+\overset{3}{\underset{i=1}{\sum }}\overset{3}{\underset{j>i}{\sum }}\beta_{ij}x_{i}x_{j}$$

where *Y* is the predicted OD value, *β*_0_ is a constant, *β_i_*, *β_ii_*, and *β_ij_* are the linear, quadratic, and interactive coefficients of the model, respectively. *X_i_* and *X_j_* are independent variables (*i* ≠ *j*).

The optimization of UAE parameters synchronously is extremely significant for predicting the extraction efficiency of eleven compounds. The desirability function approach is applied to reflect the overall effect for the optimization of multiple characteristics concurrently. According to preferred conditions of each inspection index, each index was standardized into a desirability value (*d_i_*) between 0 and 1, and the overall desirability values were obtained through calculation of the geometric mean of the *d_i_* of each index.

(3) $$0\;\leq \;d_{i}\;\leq \;1$$

(4) $$\mathrm{OD}={\left(d_{1}\times d_{2}\times \ldots \times d_{n}\right)}^{1/n}$$

where *n* is the number of indexes. The desirability value was mathematically transformed by Hassan method to obtain the smaller the better factor and the larger the better factor, respectively. If the characteristic *Y_i_* reaches its target, then *d_i_* = 1. If the characteristic is outside an acceptable region, then *d_i_* = 0.

*d_i_* = (*Y_i_
*−* Y*_min_)/(*Y*_max_ − *Y*_min_) (5)

where$\;Y_{i}$ is the actual measured value; *Y*_min_ and *Y*_max_ refer to the minimum and maximum of all values which were measured in different tests for each index, respectively.

## 4. Conclusions

This study developed a rapid and simple method for simultaneous determination of multiple components of CH based on UHPLC-variable wavelength detection, and the Box-Behnken design of UAE for optimizing the extraction conditions to maximize the extraction of components with different type of structure using 65% methanol–water at a liquid to solid ratio of 70:1 mL/g for 40.0 min. The results demonstrated that the variable wavelength detection method is suitable for CH with complex and diverse ingredients and found that the optimum harvest period was before flowering (around July). These findings can offer a new strategy for quality control of TCMs.

## Figures and Tables

**Table 1:** Analytical properties for validated UHPLC-DAD methods.

Analytes	Calibration Curves	Linear Range (μg/mL)	*R* ^2^	LOQ (μg/mL)	LOD (μg/mL)	Stability (RSD%, *n* = 6)	Precision (RSD%, *n* = 6)	Repeatability (RSD%, *n* = 6)	Average Recovery (%, *n* = 9)	Average Recovery (RSD%, *n* = 9)
**1**	*Y* = 13.984*X* − 3.0103	143.00–2.86	0.9997	0.0091	0.0046	0.85	0.75	2.10	98.52	0.62
**2**	*Y* = 14.502*X* − 4.3899	1088.00–21.74	0.9996	0.0150	0.0076	0.20	0.72	1.54	99.09	1.10
**3**	*Y* = 12.039*X* − 44.602	199.00–3.98	0.9995	0.0820	0.0270	1.24	0.74	1.97	101.74	0.87
**4**	*Y* = 17.283*X* − 34.682	195.00–3.70	0.9996	0.0170	0.0065	0.66	0.78	1.82	102.62	0.87
**5**	*Y* = 2.4643*X* − 1.4829	160.00–2.60	0.9995	0.0450	0.0180	0.45	0.70	1.57	98.51	0.42
**6**	*Y* = 6.3739*X* − 3.0096	57.00–1.14	0.9996	0.2800	0.1400	1.97	2.89	2.52	100.04	0.55
**7**	*Y* = 8.5706*X* + 1.4295	1076.00–21.52	0.9995	0.0160	0.0081	0.22	0.76	1.54	101.17	1.81
**8**	*Y* = 20.834*X* − 0.4984	70.00–1.40	0.9995	0.0270	0.0130	0.65	0.94	1.08	98.28	1.03
**9**	*Y* = 11.296*X* − 1.6736	63.00–1.26	0.9996	0.0410	0.0170	0.57	0.95	2.14	99.72	1.05
**10**	*Y* = 14.782*X* − 4.4511	101.00–1.01	0.9998	0.0140	0.0070	2.48	1.86	2.22	101.31	1.40
**11**	*Y* = 22.297*X* − 4.2959	100.00–2.00	0.9996	0.0240	0.0089	0.21	0.74	1.03	97.30	0.85

Note: *X*, concentration of standard solution, μg/mL. *Y*, the corresponding peak area.

**Table 2:** The Box-Behnken design matrix and the results for response yield (OD value) of *Clinopodium polycephalum*.

Run	*X*_1_ (Methanol–Water Proportion, %)	*X*_2_ (Liquid to Solid Ratio, mL/g)	*X*_3_ (Extraction Time, Min)	*Y* = OD
1	−1 (50)	−1 (30)	0 (35)	0.4249
2	1 (90)	−1 (30)	0 (35)	0.0000
3	−1 (50)	1 (70)	0 (35)	0.8700
4	1 (90)	1 (70)	0 (35)	0.4947
5	−1 (50)	0 (50)	−1 (20)	0.6189
6	1 (90)	0 (50)	−1 (20)	0.0000
7	−1 (50)	0 (50)	1 (50)	0.6671
8	1 (90)	0 (50)	1 (50)	0.3522
9	0 (70)	−1 (30)	−1 (20)	0.4575
10	0 (70)	1 (70)	−1 (20)	0.8231
11	0 (70)	−1 (30)	1 (50)	0.6224
12	0 (70)	1 (70)	1 (50)	0.9318
13	0 (70)	0 (50)	0 (35)	0.8038
14	0 (70)	0 (50)	0 (35)	0.8591
15	0 (70)	0 (50)	0 (35)	0.7822
16	0 (70)	0 (50)	0 (35)	0.8030
17	0 (70)	0 (50)	0 (35)	0.7969

**Table 3:** Variance analysis of response surface quadratic model.

Source	Sum of Square	DF	Mean Square	*F* Value	*p* Value	Significant
Model	1.2900	9	0.1429	60.84	<0.0001	**
*X*_1_	0.3758	1	0.3758	160.00	<0.0001	**
*X*_2_	0.3259	1	0.3259	138.76	<0.0001	**
*X*_3_	0.0568	1	0.0568	24.17	0.0017	**
*X*_1_ *X*_2_	0.0006	1	0.0006	0.26	0.6246	
*X*_1_ *X*_3_	0.0231	1	0.0231	9.84	0.0165	*
*X*_2_ *X*_3_	0.0008	1	0.0008	0.34	0.5802	
$X_{1}^{2}$	0.4596	1	0.4596	195.64	<0.0001	**
$X_{2}^{2}$	0.0041	1	0.0041	1.75	0.2277	
$X_{3}^{2}$	0.0201	1	0.0201	8.55	0.0222	*
Residual	0.0164	7	0.0023			
Lack of fit	0.0130	3	0.0043	5.04	0.0760	
Pure error	0.0034	4	0.0009			
Cor Total	1.3000	16				

Note: **, Highly significant (*p* < 0.001). *, Very significant (*p* < 0.01). Note: Coefficient of determination *R^*2*^* = 0.9874 and adjusted *R^*2*^_*adj*_* = 0.9711.

**Table 4:** Contents (mg/g) of the eleven representative components in the 24 batches of *Clinopodium polycephalum* samples.

No.	1	2	3	4	5	6	7	8	9	10	11
S1/June	2.520 ± 0.045	10.006 ± 0.249	3.878 ± 0.090	4.609 ± 0.080	5.619 ± 0.134	0.126 ± 0.005	23.821 ± 0.557	0.122 ± 0.010	0.671 ± 0.018	0.060 ± 0.001	0.337 ± 0.008
S1/July	1.503 ± 0.098	11.545 ± 0.123	5.816 ± 0.089	7.119 ± 0.018	6.206 ± 0.075	0.122 ± 0.006	27.044 ± 0.297	0.048 ± 0.002	1.853 ± 0.037	0.067 ± 0.000	0.089 ± 0.000
S1/August	1.906 ± 0.093	9.543 ± 0.272	1.672 ± 0.138	4.462 ± 0.133	3.953 ± 0.118	0.256 ± 0.006	15.169 ± 0.434	0.046 ± 0.001	1.217 ± 0.036	0.051 ± 0.001	0.154 ± 0.009
S2/June	2.505 ± 0.127	18.100 ± 0.935	3.696 ± 0.186	5.272 ± 0.271	8.138 ± 0.443	0.172 ± 0.013	35.824 ± 1.670	0.184 ± 0.017	0.448 ± 0.045	0.055 ± 0.003	0.413 ± 0.020
S2/July	1.584 ± 0.088	23.221 ± 0.671	4.052 ± 0.234	6.413 ± 0.265	6.111 ± 0.387	0.106 ± 0.008	48.868 ± 1.355	0.311 ± 0.017	1.481 ± 0.050	0.074 ± 0.002	0.711 ± 0.035
S2/August	1.016 ± 0.062	5.474 ± 0.331	2.157 ± 0.094	3.575 ± 0.134	0.819 ± 0.066	1.128 ± 0.096	12.683 ± 0.687	0.562 ± 0.011	0.134 ± 0.011	0.054 ± 0.003	0.215 ± 0.013
S3/June	1.579 ± 0.010	8.954 ± 0.083	4.731 ± 0.194	2.635 ± 0.036	5.089 ± 0.059	0.090 ± 0.003	25.190 ± 0.224	0.306 ± 0.007	1.706 ± 0.016	0.043 ± 0.000	1.509 ± 0.019
S3/July	1.405 ± 0.166	8.348 ± 0.870	4.436 ± 0.330	5.75 ± 0.449	5.184 ± 0.158	0.209 ± 0.011	27.809 ± 2.967	0.116 ± 0.009	3.189 ± 0.383	0.052 ± 0.003	0.384 ± 0.042
S3/August	2.510 ± 0.153	7.620 ± 0.471	4.419 ± 0.275	4.322 ± 0.203	1.406 ± 0.124	4.675 ± 0.218	25.324 ± 1.638	0.652 ± 0.004	0.120 ± 0.009	0.052 ± 0.002	0.546 ± 0.036
S4/June	3.601 ± 0.045	17.075 ± 0.163	4.202 ± 0.027	6.759 ± 0.080	10.382 ± 0.13	0.208 ± 0.015	32.302 ± 0.280	0.169 ± 0.011	0.841 ± 0.012	0.052 ± 0.001	0.456 ± 0.008
S4/July	3.097 ± 0.085	15.702 ± 0.431	3.417 ± 0.074	7.303 ± 0.177	9.823 ± 0.230	0.253 ± 0.011	28.860 ± 0.745	0.066 ± 0.005	0.923 ± 0.020	0.059 ± 0.002	0.076 ± 0.001
S4/August	2.366 ± 0.026	10.156 ± 0.057	3.967 ± 0.023	6.189 ± 0.018	9.255 ± 0.082	0.710 ± 0.006	19.261 ± 0.090	0.100 ± 0.002	1.429 ± 0.022	0.046 ± 0.001	0.274 ± 0.005
S5/June	3.010 ± 0.101	15.289 ± 0.487	4.704 ± 0.119	7.916 ± 0.159	10.183 ± 0.222	0.138 ± 0.024	37.880 ± 1.148	0.123 ± 0.007	0.872 ± 0.094	0.065 ± 0.000	0.344 ± 0.010
S5/July	2.164 ± 0.053	14.735 ± 0.367	4.624 ± 0.111	8.774 ± 0.163	6.859 ± 0.075	0.161 ± 0.010	35.326 ± 0.867	0.072 ± 0.003	1.446 ± 0.022	0.070 ± 0.001	0.150 ± 0.047
S5/August	1.385 ± 0.068	8.834 ± 0.938	3.492 ± 0.366	6.162 ± 0.391	7.683 ± 0.049	0.747 ± 0.048	24.080 ± 2.453	0.092 ± 0.010	0.647 ± 0.067	0.062 ± 0.003	0.410 ± 0.047
S6/June	3.250 ± 0.146	20.325 ± 0.888	5.630 ± 0.214	5.728 ± 0.180	7.655 ± 0.376	0.115 ± 0.008	35.405 ± 1.527	0.161 ± 0.005	1.26 ± 0.031	0.051 ± 0.003	0.301 ± 0.012
S6/July	1.589 ± 0.025	17.057 ± 0.208	3.723 ± 0.047	5.411 ± 0.099	6.784 ± 0.033	0.138 ± 0.013	33.067 ± 0.381	0.131 ± 0.003	2.280 ± 0.020	0.063 ± 0.000	0.236 ± 0.003
S6/August	1.425 ± 0.020	8.137 ± 0.075	2.705 ± 0.012	4.673 ± 0.059	7.977 ± 0.146	0.395 ± 0.001	14.077 ± 0.150	0.100 ± 0.004	1.483 ± 0.014	0.063 ± 0.000	0.188 ± 0.002
S7/June	1.413 ± 0.025	9.459 ± 0.098	4.589 ± 0.058	4.060 ± 0.061	7.200 ± 0.098	0.102 ± 0.006	23.080 ± 0.269	0.089 ± 0.001	1.533 ± 0.031	0.055 ± 0.001	0.220 ± 0.004
S7/July	0.637 ± 0.015	6.241 ± 0.176	2.655 ± 0.062	3.788 ± 0.133	4.341 ± 0.118	0.115 ± 0.004	19.078 ± 0.519	0.091 ± 0.006	1.880 ± 0.048	0.045 ± 0.001	0.485 ± 0.011
S7/August	1.200 ± 0.063	9.643 ± 0.460	3.632 ± 0.106	5.167 ± 0.166	6.812 ± 0.143	0.502 ± 0.017	27.336 ± 1.267	0.162 ± 0.013	1.382 ± 0.068	0.064 ± 0.002	0.553 ± 0.022
S8/June	2.149 ± 0.112	18.056 ± 0.987	3.824 ± 0.162	6.046 ± 0.253	9.047 ± 0.199	0.308 ± 0.011	29.040 ± 1.603	0.145 ± 0.007	0.618 ± 0.027	0.063 ± 0.002	0.282 ± 0.017
S8/July	1.038 ± 0.019	9.945 ± 0.165	3.337 ± 0.124	4.835 ± 0.081	5.060 ± 0.087	0.205 ± 0.016	29.470 ± 0.434	0.095 ± 0.005	1.120 ± 0.043	0.067 ± 0.001	0.336 ± 0.007
S8/August	1.345 ± 0.029	8.745 ± 0.328	2.886 ± 0.064	5.338 ± 0.130	7.058 ± 0.102	0.579 ± 0.006	18.646 ± 0.682	0.073 ± 0.013	1.357 ± 0.062	0.056 ± 0.001	0.200 ± 0.010

Note: No. **1**, **2**, **3**, **4**, **5**, **6**, **7**, **8**, **9**, **10** and **11** represent the eleven representative components in the order of retention time. All data were performed in parallel three times (Mean ± SD, *n* = 3).

**Table 5:** The level of independent variables for Box-Behnken design.

Independent Variables	Levels		
	−1	0	1
Methanol–water proportion (X1) (%)	50	70	90
Liquid to solid ratio (X2) (mL/g)	30	50	70
Extraction time (X3) (min)	20	35	50

## Data Availability

The data presented in this study are available in the Supplementary Materials.

## Supplementary Materials

The following supporting information can be downloaded at: https://www.mdpi.com/article/10.3390/molecules27144418/s1.

## References

[1] Normile D. (2003). The new face of traditional Chinese medicine. Science.

[2] Yao C.L., Zhang J.Q., Li J.Y., Wei W.L., Wu S.F., Guo D.A. (2021). Traditional Chinese medicine (TCM) as a source of new anticancer drugs. Nat. Prod. Rep..

[3] Jiang Y., David B., Tu P., Barbin Y. (2010). Recent analytical approaches in quality control of traditional Chinese medicines—A review. Anal. Chim. Acta.

[4] Xie P.S., Leung A.Y. (2009). Understanding the traditional aspect of Chinese medicine in order to achieve meaningful quality control of Chinese materia medica. J. Chromatogr. A.

[5] Li H., Wang S.W., Zhang B.L., Xie Y.H., Yang Q., Cao W., Wang J.B. (2011). Simultaneous quantitative determination of 9 active components in traditional Chinese medicinal preparation ShuangDan oral liquid by RP-HPLC coupled with photodiode array detection. J. Pharm. Biomed. Anal..

[6] Wang H., Jiang Y., Ding M., Li J., Hao J., He J., Wang H., Gao X., Chang Y. (2018). Simultaneous determination and qualitative analysis of six types of components in Naoxintong capsule by miniaturized matrix solid-phase dispersion extraction coupled with ultra-high performance liquid chromatography with photodiode array detection and quadrupole time-of-flight mass spectrometry. J. Sep. Sci..

[7] Murata T., Sasaki K., Sato K., Yoshizaki F., Yamada H., Mutoh H., Umehara K., Miyase T., Warashina T., Aoshima H. (2009). Matrix Metalloproteinase-2 Inhibitors from *Clinopodium chinense* var. parviflorum. J. Nat. Prod..

[8] Zhong M., Sun G., Zhang X., Sun G., Xu X., Yu S. (2012). A New Prenylated Naphthoquinoid from the Aerial Parts of *Clinopodium chinense* (Benth.) O. Kuntze. Molecules.

[9] Zhong M., Wu H., Zhang X., Sun G., Yu S., Xu X. (2014). A new diterpene from *Clinopodium chinense*. Nat. Prod. Res..

[10] Zhu Y.D., Wu H.F., Ma G.X., Chen R.C., Long H.L., Zuo Z.L., Luo Y., Zhu N.L., Hou B., Xu X.D. (2016). Clinoposides A–F: Meroterpenoids with protective effects on H9c2 cardiomyocyte from *Clinopodium chinense*. RSC Adv..

[11] Zhu Y.D., Chen R.C., Wang H., Jiang H., Huang X.L., Zhang M.L., Li L.Y., Hu Z., Xu X.D., Wang C.J. (2018). Two new flavonoid–triterpene saponin meroterpenoids from *Clinopodium chinense* and their protective effects against anoxia/reoxygenation-induced apoptosis in H9c2 cells. Fitoterapia.

[12] Li L., Huang Q., Duan X., Han L., Peng D. (2020). Protective effect of *Clinopodium chinense* (Benth.) O. Kuntze against abnormal uterine bleeding in female rats. J. Pharmacol. Sci..

[13] Zeng B., Liu G.D., Zhang B.B., Wang S., Ma R., Zhong B.S., He B., Liang Y., Wu F.H. (2020). A new triterpenoid saponin from *Clinopodium chinense* (Benth.) O. Kuntze. Nat. Prod. Res..

[14] Zhu Y.D., Hong J.Y., Bao F.D., Xing N., Wang L.T., Sun Z.H., Luo Y., Jiang H., Xu X.D., Zhu N.L. (2017). Triterpenoid saponins from *Clinopodium chinense* (Benth.) O. Kuntze and their biological activity. Arch. Pharmacal Res..

[15] Shi X., Wang S., Luan H., Tuerhong D., Lin Y., Liang J., Xiong Y., Rui L., Wu F. (2019). *Clinopodium chinense* Attenuates Palmitic Acid-Induced Vascular Endothelial Inflammation and Insulin Resistance through TLR4-Mediated NF-κB and MAPK Pathways. Am. J. Chin. Med..

[16] Li J., Wu F.H., Su J.B., Ma S.P. (2012). Study on antioxidation of active fraction from *Clinopodium Chinese* in vitro. Strait Pharm. J..

[17] Gao Y., Wang Y., Wang K., Zhu J., Li G., Tian J., Li C., Wang Z., Li J., Lee A.W. (2017). Acute and a 28-day repeated-dose toxicity study of total flavonoids from *Clinopodium chinense* (Benth.) O. Ktze in mice and rats. Regul. Toxicol. Pharmacol..

[18] Zhang H.J., Chen R.C., Sun G.B., Yang L.P., Zhu Y.D., Xu X.D., Sun X.B. (2018). Protective effects of total flavonoids from *Clinopodium chinense* (Benth.) O. Ktze on myocardial injury in vivo and in vitro via regulation of Akt/Nrf2/HO-1 pathway. Phytomedicine.

[19] Committee Chinese Pharmacopoeia (2020). Pharmacopoeia of the People’s Republic of China.

[20] Wu W.B., Zhao C. (2005). Determing the content of hesperidin in *Clinopodium polycephalum* by HPLC. Res. Pract. Chin. Med..

[21] He B., Tian J., Li C.H., Ai H.B. (2008). SPE-HPLC determination of buddlejasaponinsIVb in Duanxueliu capsules. Chin. J. Pharm. Anal..

[22] Huang Y., Yuan X.H., Liu Z. (2014). HPLC Determination of didymin in duanxueliu Herb. J. Anhui Agric. Sci..

[23] Kang N., Gao X.Y., Fan Q.L., Lin Y., Xiao W. (2015). Determination of Clinodiside A in Clinopodium herb by HPLC. J. China Prescr. Drug.

[24] Lin F.Y., Sun Y.X., Chen R.Y. (2020). Determination of Clinodiside A in Clinopodium oral liquids by HPLC. J. Pharm. Res..

[25] Ren X., Shao X.X., Li X.X., Jia X.H., Song T., Zhou W.Y., Wang P., Li Y., Wang X.L., Cui Q.H. (2020). Identifying potential treatments of COVID-19 from Traditional Chinese Medicine (TCM) by using a data-driven approach. J. Ethnopharmacol..

[26] Esmaeili F., Hashemiravan M., Eshaghi M.R., Gandomi H. (2021). Optimization of Aqueous Extraction Conditions of Inulin from the *Arctium lappa* L. Roots Using Ultrasonic Irradiation Frequency. J. Food Qual..

[27] Alara O.R., Abdurahman N.H., Ukaegbu C.I. (2021). Extraction of phenolic compounds: A review. Curr. Res. Food Sci..

[28] Vuong Q.V., Nguyen V.T., Thanh D.T., Bhuyan D.J., Goldsmith C.D., Sadeqzadeh E., Scarlett C.J., Bowyer M.C. (2015). Optimization of ultrasound-assisted extraction conditions for euphol from the medicinal plant, *Euphorbia tirucalli*, using response surface methodology. Ind. Crop. Prod..

[29] Jagadeesan G., Muniyand K., Lydia M.A., Nataraj G., Thamburaj S., Sathyanarayanan S., Thangaraj P. (2020). Analysis of discrete and combined effect of solvent, extraction time, and extraction temperature on polyphenol compounds extraction from roxburgh fig (*Ficus auriculata* Lour.) fruit using response surface methodology. Phytomedicine Research and Development.

[30] Subramani T., Ganapathyswamy H., Sampathrajan V., Sundararajan A. (2021). Optimization of extraction parameters to improve cottonseed milk yield and reduce gossypol levels using response surface methodology. J. Food Process. Preserv..

[31] Wang K.H., Li G.Q., Li K.M., Naumovski V.R., Chan K. (2017). Optimisation of Pueraria isoflavonoids by response surface methodology using ultrasonic-assisted extraction. Food Chem..

[32] Elboughdiri N., Ghernaout D., Kriaa K., Jamoussi B. (2020). Enhancing the extraction of phenolic compounds from Juniper Berries using the box-behnken design. ACS Omega.

[33] Kowalska I., Adach W., Stochmal A., Olas B. (2020). A comparison of the effects of apigenin and seven of its derivatives on selected biomarkers of oxidative stress and coagulation in vitro. Food Chem. Toxicol..

[34] Xie M.J., Yu Y.T., Zhu Z.Y., Deng L.P., Ren B., Zhang M. (2021). Simultaneous determination of six main components in Bushen Huoxue prescription by HPLC-CAD. J. Pharm. Biomed. Anal..

[35] Qiu Y.X., Huang J.H., Jiang X.M., Chen Y., Liu Y., Zeng R., Shehla N., Liu Q., Liao D.F., Guo D.A. (2015). Quantitative and qualitative determination of LiuweiDihuang preparations by ultra-high-performance liquid chromatography in dual-wavelength fingerprinting mode and random forest. J. Sep. Sci..

[36] Duan L., Guo L., Liu K., Liu E.H., Li P. (2014). Characterization and classification of seven Citrus herbs by liquid chromatography–quadrupole time-of-flight mass spectrometry and genetic algorithm optimized support vector machines. J. Chromatogr. A.

[37] Miladi M., Martins A.A., Mata T.M., Vegara M., Pérez-Infantes M., Remmani R., Ruiz-Canales A., Núñez-Gómez D. (2021). Optimization of Ultrasound-Assisted Extraction of Spent Coffee Grounds Oil Using Response Surface Methodology. Processes.

[38] Halim R., Gladman B., Danquah M.K., Webley P.A. (2011). Oil extraction from microalgae for biodiesel production. Bioresour. Technol..

[39] Mason T.J., Paniwnyk L., Lorimer J.P. (1996). The uses of ultrasound in food technology. Ultrason. Sonochem..

[40] Wu Y., Wang X., Fan E. (2011). Optimisation of ultrasound-assisted extraction of puerarin and total isoflavones from *Puerariae Lobatae* Radix (Pueraria lobata (Wild.) Ohwi) with response surface methodology. Phytochem. Anal..

[41] Cheok C.Y., Chin N.L., Yusof Y.A., Talib R.A., Law C.L. (2012). Optimization of total phenolic content extracted from *Garcinia mangostana* Linn. hull using response surface methodology versus artificial neural network. Ind. Crops Prod..

