# Supplementary material for: A Rapid and Efficient Strategy for Quality Control of Clinopodii herba Encompassing Optimized Ultrasound-Assisted Extraction Coupled with Sensitive Variable Wavelength Detection
Source: Molecules. 2022 Jul 10;27(14):4418. doi: 10.3390/molecules27144418 (PMC9316769; doi:10.3390/molecules27144418)
Supplement: Supplementary file 1 [file molecules-27-04418-s001.zip › molecules-1764646-supplementary.pdf]

Supplementary Materials

A Rapid and Efficient Strategy for Quality Control of Clinopodii herba Encompassing Optimized Ultrasound-Assisted Extraction Coupled with Sensitive Variable Wavelength Detection

Yao Liu [1,†], Xiaojun Song [1,2,†], Xuebin Shen [1], Yuangen Xiong [3], Li Liu [1], Yuexi Yang [1], Sihui Nian [1,2,*] and Limin Liu [4,*]

1. School of Pharmacy, Wannan Medical College, Wuhu 241002, China; liuyao@stu.wnmc.edu.cn (Y.L.); tsongxj@wnmc.edu.cn (X.S.); sxbchn@wnmc.edu.cn (X.S.); liulilili@stu.wnmc.edu.cn (L.L.); yx0603@stu.wnmc.edu.cn (Y.Y.); niansihui@wnmc.edu.cn (S.N.)
2. Institute of Modern Chinese Medicine, Wannan Medical College, Wuhu 241002, China; tsongxj@wnmc.edu.cn (X.S.); niansihui@wnmc.edu.cn (S.N.)
3. Pharmaceutical Co., Ltd., Huiyinbi Group Co., Ltd., Lu'an 237202, xyg19640806@163.com (Y.X)
4. Anhui College of Traditional Chinese Medicine, Wuhu 241000, China; liulm2005@ahzyygz.edu.cn (L.L.)

† Correspondence: authors contributed equally to this work.

* Correspondence: niansihui@wnmc.edu.cn (S.N.); liulm2005@ahzyygz.edu.cn (L.L.)

Table S1. Chemical structures and UV spectra of eleven compounds.

Compound	Chemical Structure	UV Spectrum
1	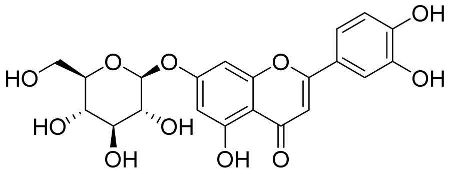	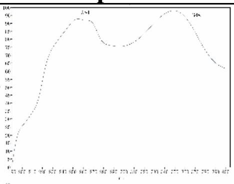
2	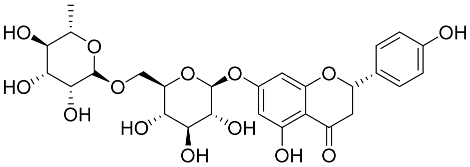	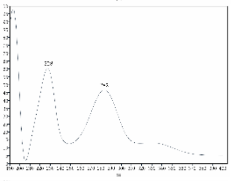
3	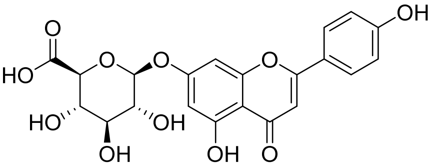	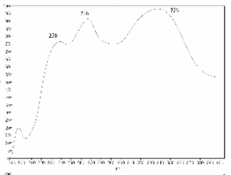
4	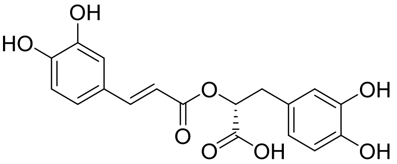	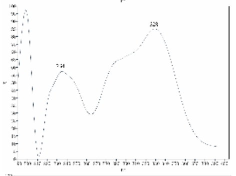
5	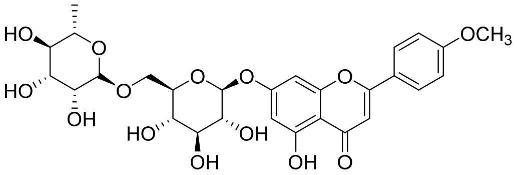	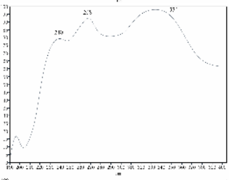
6	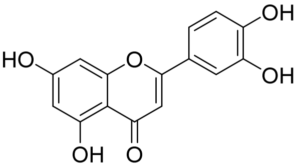	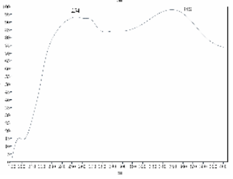

**7**	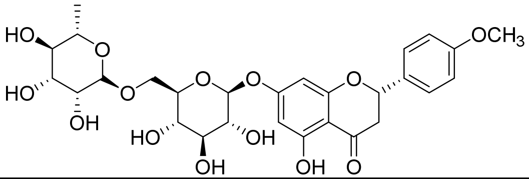	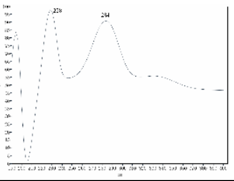
**8**	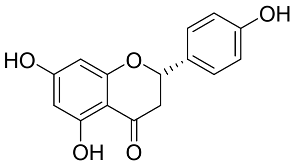	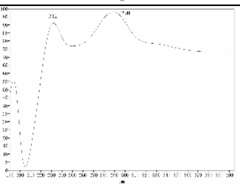
**9**	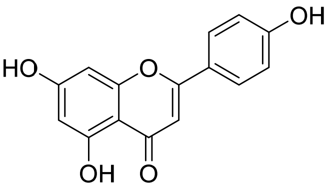	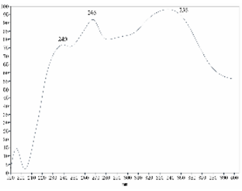
**10**	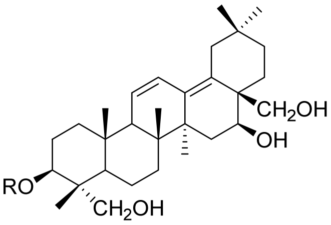 R = [β-D-Glc-(1-2)]-[β-D-Glc-(1-3)]-β-D-Fuc	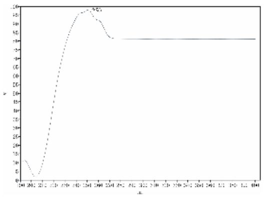
**11**	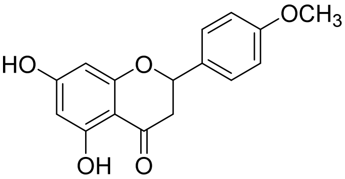	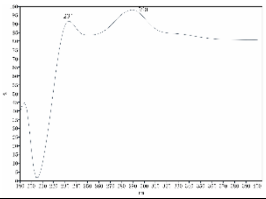

Table S2. Effects of different extraction parameters on the extraction efficiency of eleven components.

Independent Variables	Factor Level	Concentrations (mg/g ± SD) (*n* = 3)											OD value
		**1**	**2**	**3**	**4**	**5**	**6**	**7**	**8**	**9**	**10**	**11**	
X_1: methanol-water (%)	40	1.813±0.014	10.045±0.252	3.689±0.015	2.278±0.035	4.978±0.032	0.538±0.006	21.943±0.554	0.576±0.035	0.140±0.001	0.115±0.000	2.526±0.038	0.000±0.000
	50	1.854±0.002	10.416±0.030	3.764±0.003	2.222±0.014	5.607±0.039	0.560±0.006	23.258±0.171	0.925±0.018	0.176±0.016	0.124±0.000	3.593±0.105	0.497±0.069
	60	1.877±0.033	10.573±0.315	3.798±0.049	2.430±0.007	6.139±0.018	0.629±0.011	24.442±0.785	1.009±0.137	0.202±0.005	0.126±0.001	3.660±0.598	0.775±0.034
	70	1.871±0.008	10.786±0.167	3.810±0.023	2.413±0.049	6.245±0.005	0.637±0.016	24.925±0.236	1.016±0.027	0.201±0.009	0.128±0.000	3.740±0.029	0.960±0.007
	80	1.696±0.002	10.889±0.058	3.640±0.017	2.386±0.009	5.96±0.042	0.525±0.017	25.599±0.141	0.886±0.000	0.193±0.001	0.125±0.001	3.101±0.003	0.760±0.008
	90	1.495±0.016	9.877±0.197	3.208±0.030	2.223±0.010	4.910±0.048	0.280±0.001	23.184±0.563	0.983±0.033	0.176±0.015	0.119±0.002	3.600±0.172	0.186±0.116
X_2: liquid to solid ratio (mL/g)	30	1.673±0.006	11.315±0.013	3.448±0.007	2.339±0.006	5.903±0.025	0.612±0.004	26.276±0.016	0.637±0.010	0.208±0.013	0.106±0.000	1.908±0.006	0.000±0.000
	40	1.769±0.035	11.638±0.164	3.644±0.060	2.437±0.043	6.155±0.133	0.648±0.015	27.207±0.416	0.668±0.012	0.232±0.006	0.113±0.002	1.964±0.030	0.397±0.263
	50	1.805±0.027	11.951±0.094	3.754±0.035	2.495±0.026	6.238±0.137	0.658±0.013	27.961±0.220	0.666±0.010	0.224±0.008	0.118±0.001	2.007±0.022	0.767±0.123
	60	1.824±0.014	11.951±0.126	3.802±0.051	2.536±0.037	6.339±0.106	0.664±0.019	27.414±0.335	0.661±0.010	0.216±0.023	0.120±0.002	2.015±0.031	0.724±0.201
	70	1.780±0.004	11.728±0.031	3.862±0.009	2.511±0.010	6.175±0.027	0.641±0.010	27.311±0.089	0.646±0.001	0.223±0.001	0.125±0.000	1.994±0.000	0.734±0.210
X_3: extraction time (min)	5	1.644±0.036	11.334±0.048	3.472±0.062	2.350±0.017	5.718±0.075	0.588±0.006	26.235±0.136	0.608±0.011	0.191±0.007	0.103±0.008	1.898±0.025	0.000±0.000
	15	1.633±0.029	11.407±0.110	3.603±0.021	2.392±0.016	5.864±0.062	0.614±0.003	26.556±0.209	0.645±0.006	0.217±0.000	0.114±0.001	1.925±0.018	0.397±0.208
	25	1.736±0.006	11.551±0.006	3.717±0.015	2.426±0.007	6.019±0.005	0.628±0.001	26.824±0.010	0.634±0.000	0.218±0.004	0.114±0.000	1.940±0.001	0.619±0.113
	35	1.769±0.008	11.613±0.083	3.773±0.019	2.450±0.010	6.138±0.057	0.639±0.001	27.223±0.252	0.652±0.017	0.218±0.002	0.116±0.000	1.957±0.001	0.873±0.093
	45	1.785±0.041	11.758±0.105	3.855±0.073	2.494±0.023	6.274±0.147	0.641±0.009	27.092±0.128	0.659±0.029	0.218±0.003	0.114±0.005	1.981±0.010	0.738±0.202

Table S3. Optimum conditions and the predicted and experimental value of response at the optimum conditions (Mean ± SD, *n*=6).

	**Proportion of Methanol-Water (%)**	**Liquid to Solid Ratio (*v*/*w*)**	**Extraction Time (min)**	**Extraction Temperature (°C)**	**OD Value**
Optimum condition (predicted)	64.63	70:1	40.4	63.61	1.02
Modified condition (actual)	65	70:1	40	64.0	0.99±0.013

Table S4. The origin information of 24 batches of materials.3.

**Samples**	**Collecting Place**	**Collecting Time**	**Voucher Number**
S1	Jinzhai county N31°24′, E115°45′	June	20210613-1
		July	20210720
		August	20210821-1
S2	Jinzhai county N31°20′, E115°57′	June	20210613-2
		July	20210719-1
		August	20210821-2
S3	Huoshan county N31°39′, E116°33′	June	20210614-1
		July	20210718-1
		August	20210823
S4	Huoshan county N31°07′ E116°00′	June	20210614-2
		July	20210719-2
		August	20210822-1
S5	Yingshan county N31°27′, E115°58′	June	20210614-3
		July	20210719-3
		August	20210822-2
S6	Huoshan county N31°50′, E115°56′	June	20210614-4
		July	20210719-4
		August	20210822-3
S7	Huoshan county N31°11′, E116°00′	June	20210614-5
		July	20210718-2
		August	20210822-4
S8	Huoshan county N31°15′, E116°11′	June	20210614-6
		July	20210718-3
		August	20210821-3

Table S5. Purities and types of eleven standard substances.

**Compounds**	**Type**	**Purity**
Cynaroside (**1**)	flavonid	99.77%
Narirutin (**2**)	flavonoid	99.76%
Apigenin-7-*O*-*β*-D-glucuronide (**3**)	flavonoid	99.01%
Rosmarinic acid (**4**)	phenylpropanoid	99.02%
Buddleoside (**5**)	flavonid	99.35%
Luteolin (**6**)	flavonid	99.55%
Isosakuranetin-7-*O*-rutinoside (**7**)	flavonid	99.85%
Naringenin (**8**)	flavonid	99.78%
Apigenin (**9**)	flavonid	99.42%
Buddlejasaponin IVb (**10**)	triterpenoid saponin	99.32%
Isosakuranetin (**11**)	flavonid	99.00%

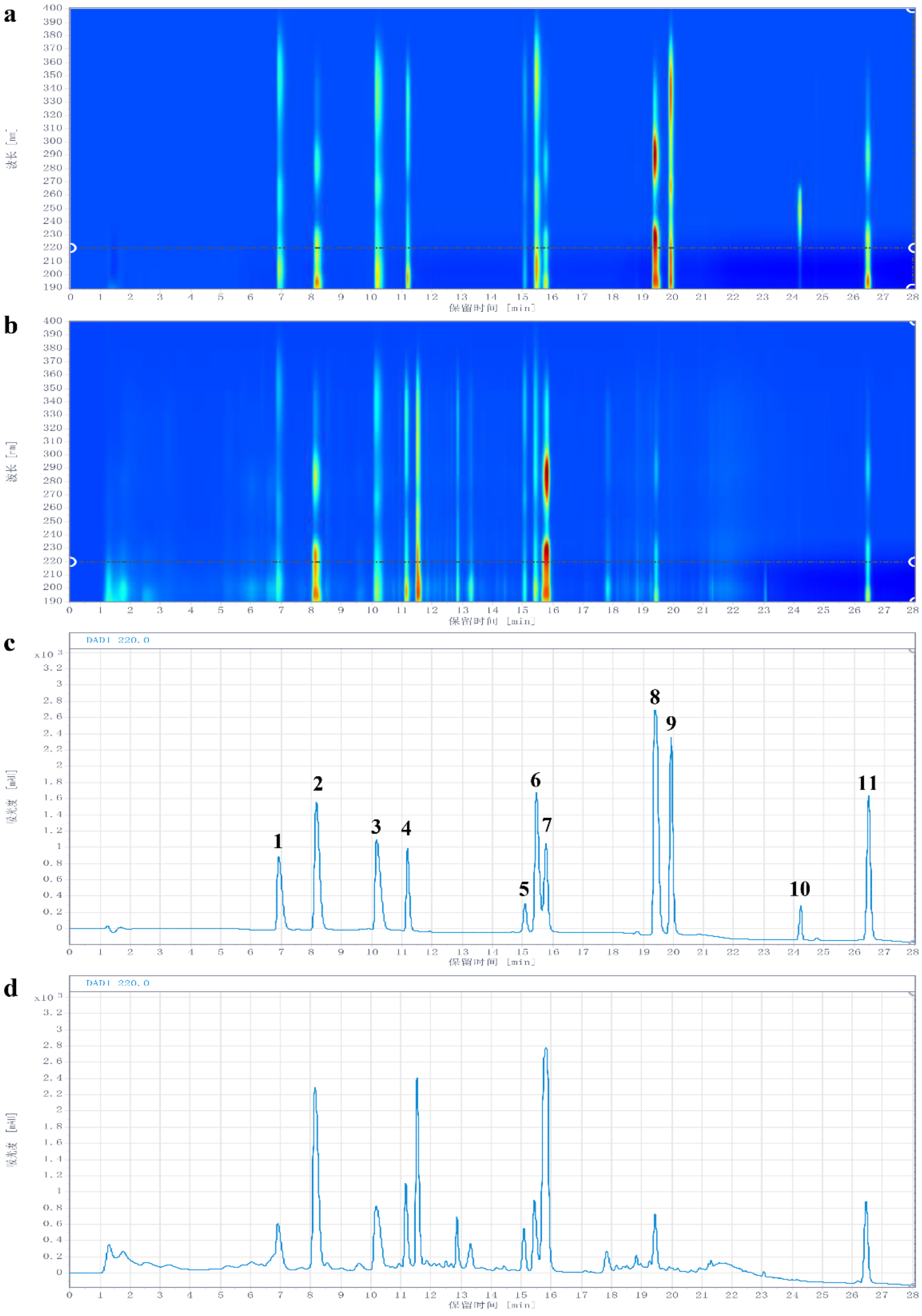

Figure S1. The Chemical fingerprint of the 70% ethyl alcohol extract of *Clinopodium polycephalum*. **a.** The Contour plot of DAD spectrums of eleven standard substances; **b.** The Contour plot of DAD spectrums of CH. **c.** The UHPLC spectrums of eleven standard substances at 220 nm. **d.** T The UHPLC spectrums of CH at 220 nm.

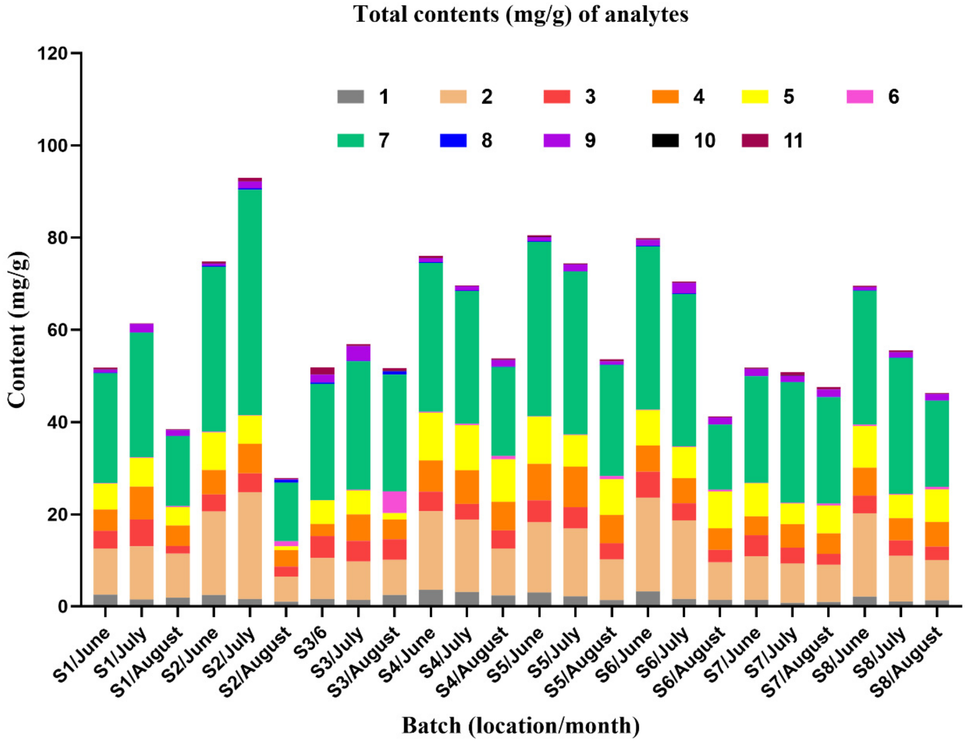

Figure S2. Total contents of the eleven compounds in *Clinopodium polycephalum* samples at different growth periods.

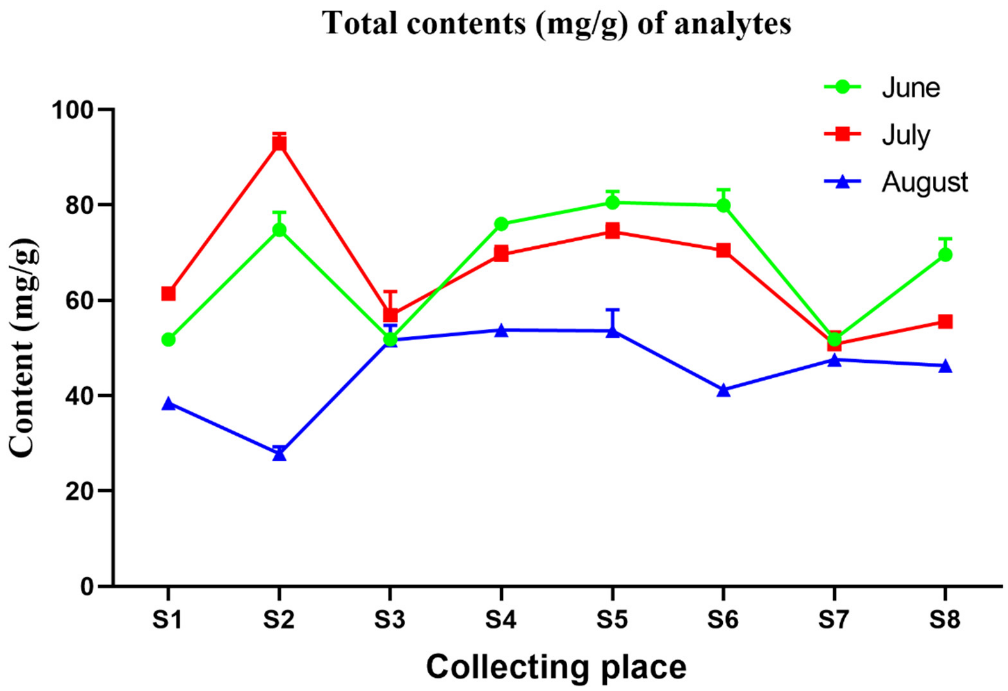

Figure S3. The total contents of eleven compounds in *Clinopodium polycephalum* samples at different growth periods. Each line represents a month, respectively.

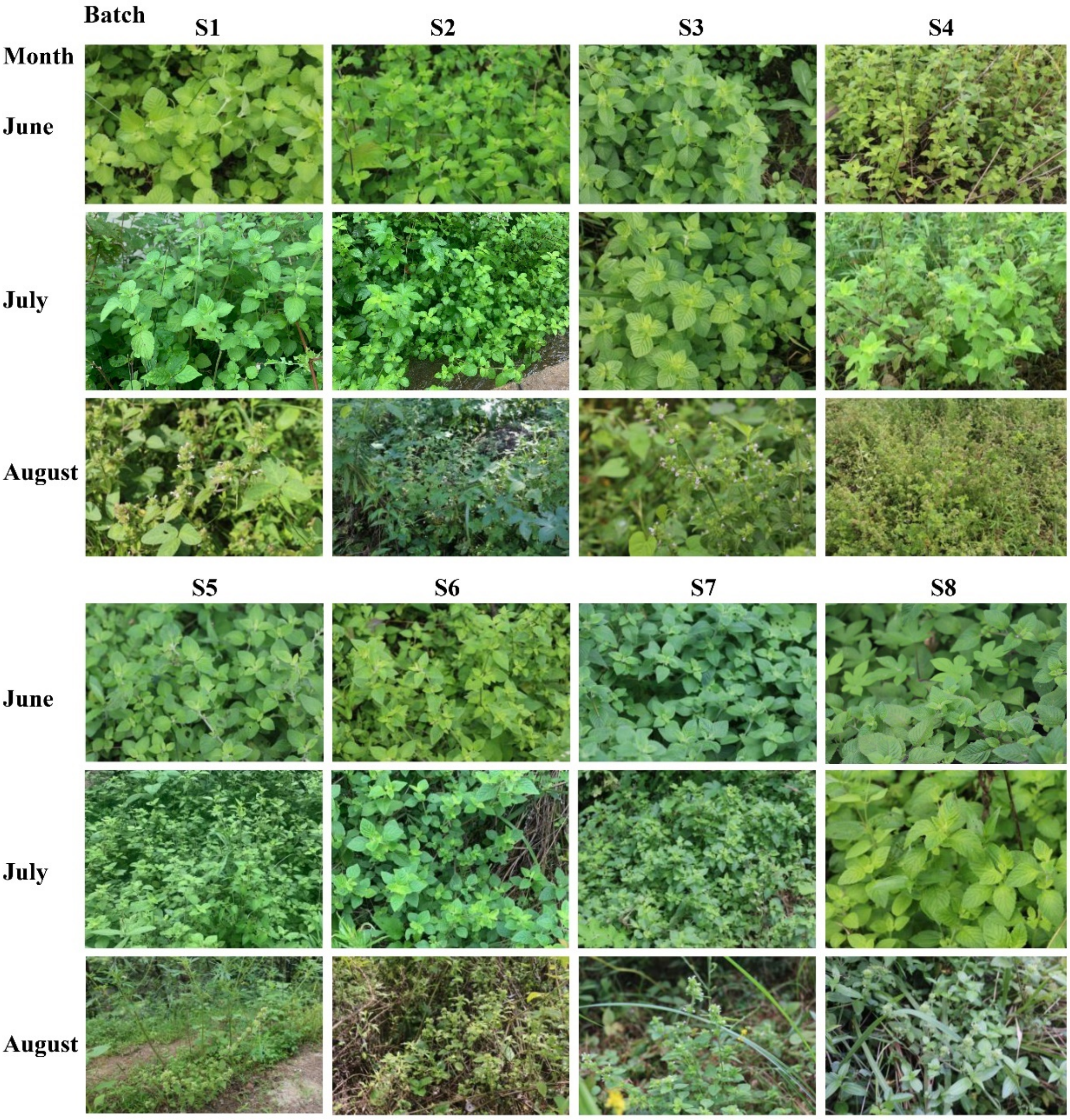

Figure S4. The original drawing of 24 batches of the different developmental stage of harvested materials.

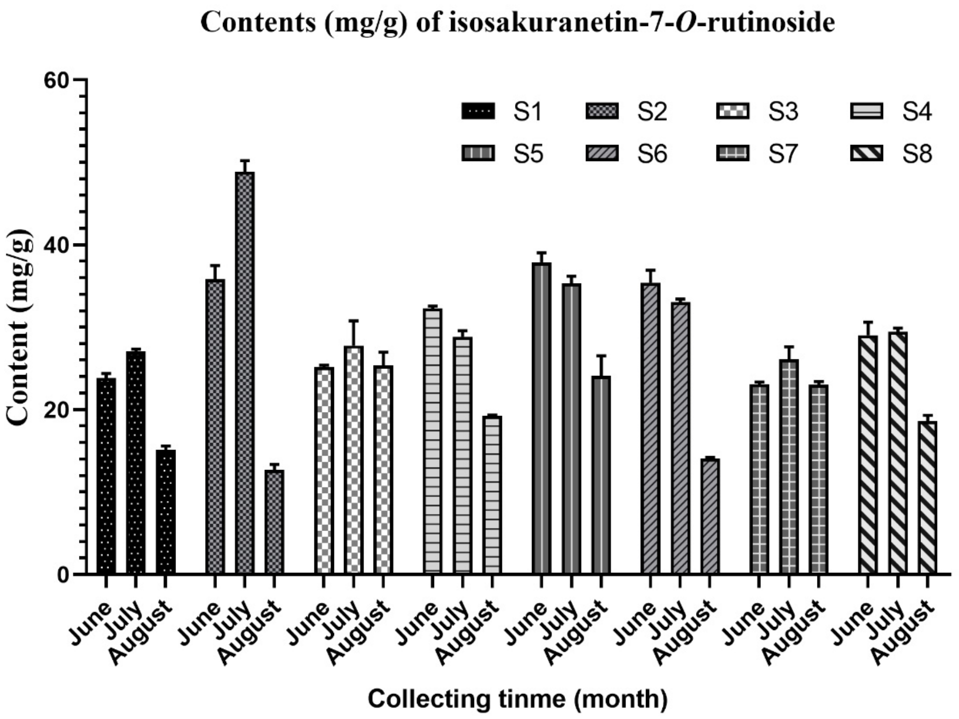

Figure S5. Dynamic accumulation of the contents of isosakuranetin-7-*O*-rutinoside in *Clinopodium polycephalum* samples at different growth periods.

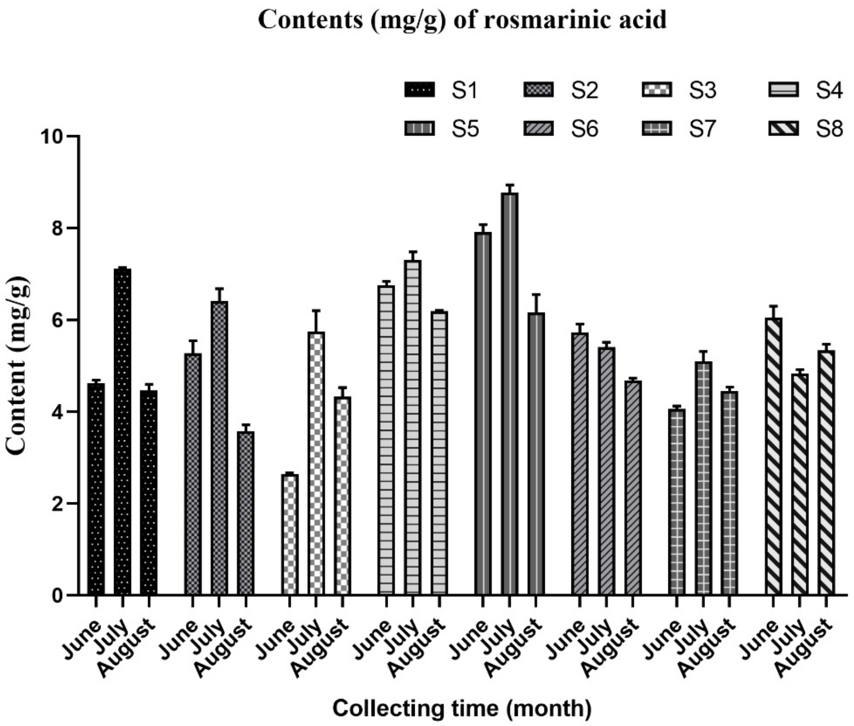

Figure S6. Dynamic accumulation of the contents of rosmarinic acid in *Clinopodium polycephalum* samples at different growth periods.

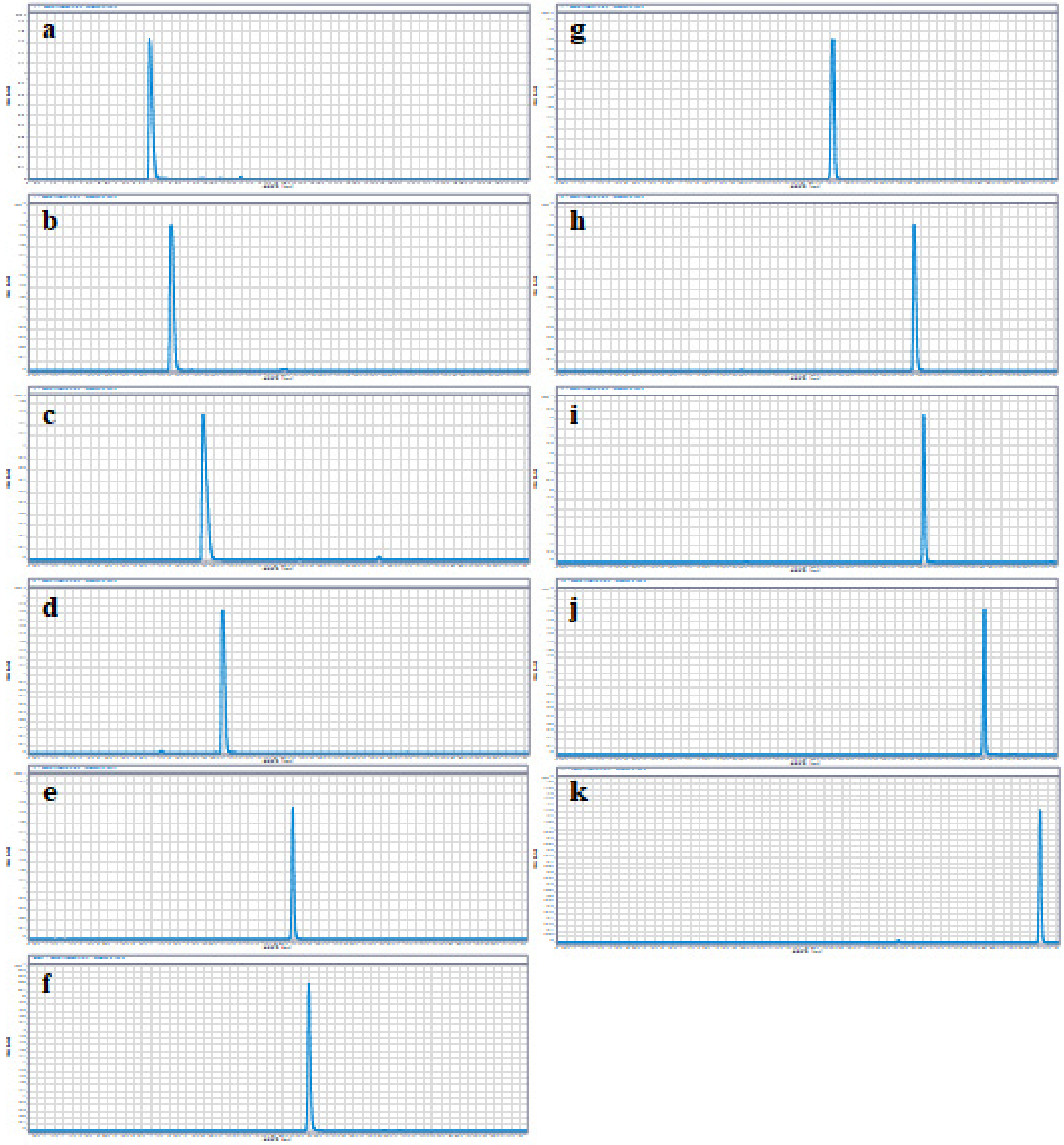

Figure S7. UHPLC spectrums of eleven standard substances. Compound **1** (**a**), Compound **2** (**b**), Compound **3** (**c**), Compound **4** (**d**), Compound **5** (**e**), Compound **6** (**f**), Compound **7** (**g**), Compound **8** (**h**), Compound **9** (**i**), Compound **10** (**j**) and Compound **11** (**k**).
